# Malnutrition assessed by the mini nutritional assessment is associated with overall survival in patients with resectable non–small cell lung cancer

**DOI:** 10.3389/fnut.2026.1864710

**Published:** 2026-07-30

**Authors:** Denis I. Trufa, Koblandy Khamitov, Lena Kopplin, Horia Sirbu

**Affiliations:** 1 Department of Thoracic Surgery, Universitätsklinikum Erlangen, Erlangen, Germany; 2 Faculty of Medicine, Friedrich-Alexander-Universität Erlangen-Nürnberg, Erlangen, Germany

**Keywords:** malnutrition, mini nutritional assessment, non–small cell lung cancer, overall survival, prognosis, pulmonary resection

## Abstract

**Background:**

Malnutrition is common in non-small cell lung cancer (NSCLC) patients undergoing surgery and is associated with poor clinical outcomes. This study aimed to assess the prevalence and prognostic value of malnutrition using the Mini Nutritional Assessment (MNA) screening tool for patient survival.

**Methods:**

A total of 98 NSCLC patients (62.2% male, median age 67 years, range: 41–84) who underwent pulmonary resection between 2012 and 2019 were evaluated for nutritional status using MNA and BMI. Overall survival (OS) was analyzed using Kaplan-Meier curves and Cox regression models. Post-surgery complications were segmented according to MNA status.

**Findings:**

According to MNA, 66.3% of patients were well-nourished (MNA ≥ 24), 31.6% were at risk of malnutrition (MNA 17–23.5), and 2% were malnourished (MNA < 17). In patients at risk of malnutrition, 1-year OS was 67.86%, and 5-year OS was 7.14%. In contrast, well-nourished patients showed a 1-year OS of 73.77%, and a 5-year OS of 26.22%. Cox regression analysis showed that MNA was significantly associated with overall survival (HR = 0.214, 95% CI 0.060–0.765, *p* = 0.018).

**Conclusion:**

Nearly one-third of NSCLC patients were at risk of malnutrition and had a significantly worse survival outcome. MNA was independently associated with overall survival, emphasizing the importance of nutritional screening in this high-risk group.

## 1 Introduction

Malnutrition is a common issue among cancer patients, affecting up to 80% of patients, depending on the type of tumor (1). It has been associated with adverse clinical outcomes, including an increased risk of postoperative complications, longer recovery times, and reduced survival rates (2). Among patients with non-small cell lung cancer (NSCLC), preoperative malnutrition is particularly critical as it can significantly affect the prognosis and overall treatment outcomes (3). Despite its importance, the detection and management of malnutrition in this patient group is often overlooked, and the reliance on simple measures such as body mass index (BMI) alone can underestimate the true extent of malnutrition.

The Mini Nutritional Assessment (MNA) is a validated and reliable screening tool that has been widely used to assess the nutritional status of elderly and cancer patients (4–6). This tool helps identify patients at risk of malnutrition by evaluating factors such as dietary intake, weight loss, and physical condition. While BMI is a useful indicator of body weight, it fails to capture the nuanced nutritional deficiencies that may be present in patients with cancer. Therefore, a more comprehensive approach to nutritional assessment is necessary to ensure better patient management and outcomes.

This study aims to evaluate the nutritional status of NSCLC patients prior to lung resection using the MNA and to explore the potential link between nutritional status and survival outcomes. By identifying patients at risk of malnutrition, it is possible to implement early interventions that may improve clinical outcomes and survival rates.

## 2 Materials and methods

### 2.1 Patient selection

Our study included patients with non-small cell lung cancer (NSCLC) who underwent pulmonary resection with curative intent between 2012 and 2019. Patients were consecutively and prospectively enrolled. Clinical characteristics were obtained from clinical records, while documented survival status and follow-up data were retrieved from the Comprehensive Cancer Center (CCC) registry according to institutional standards. The present study represents a retrospective analysis of this prospectively maintained cohort.

Inclusion criteria were as follows: (1) age ≥ 18 years, (2) histologically confirmed non-small cell lung cancer (NSCLC), (3) pulmonary resection performed with curative intent, (4) completed preoperative Mini Nutritional Assessment (MNA), and (5) written informed consent for study participation.

Patients with early-stage disease (UICC stages I–II) were routinely included. Selected patients with stage III disease and oligometastatic stage IV disease were also eligible if they were considered suitable for curative-intent surgical treatment following multidisciplinary tumor board evaluation.

The preoperative evaluation of NSCLC patients included a thorough review of demographic characteristics and tumor histology. Patients were staged at initial diagnosis according to the 8th edition of the TNM classification system issued by the International Association for the Study of Lung Cancer (IASLC) and categorized into the corresponding UICC stages. Each case was discussed by a multidisciplinary tumor board including thoracic surgeons, oncologists, and radiologists to determine the optimal treatment strategy. The clinical work-up consisted of detailed medical history, physical examination, laboratory diagnostics, and radiological staging. Cardiorespiratory fitness was assessed preoperatively using body plethysmography, transthoracic echocardiography (TTE), and electrocardiogram (ECG) in all surgical candidates.

The study protocol was approved by the Ethics Committee of the Friedrich-Alexander-University of Erlangen, Germany (Re-No: 56_12B; DRKS-ID: DRKS00005376), and written informed consent was obtained from all patients.

### 2.2 Nutritional assessment

Nutritional status was a key focus of preoperative assessment. All patients were evaluated using the Mini Nutritional Assessment (MNA), a validated screening tool consisting of 18 items. These include anthropometric measurements, global assessment, dietary questions, and subjective self-assessment by the patient. Based on the total MNA score, patients were classified as well-nourished ( ≥ 24 points), at risk of malnutrition (17–23.5 points) and malnourished ( < 17 points).

MNA evaluation was complemented by Body Mass Index (BMI), Serum albumin, C-reactive protein (CRP), and Serum creatinine. These parameters were included due to their established relevance for postoperative outcome and survival in order to prevent bias from omitted variables.

### 2.3 Statistical analysis

Statistical analyses were performed using IBM SPSS Statistics version 28 (IBM Corp., Armonk, NY, USA). Survival outcomes were analyzed using Kaplan–Meier survival analysis and compared using the log-rank test. Cox proportional hazards regression was employed to evaluate the association of nutritional and clinical variables with overall survival. Overall survival was defined as the time from pulmonary resection to death from any cause or last follow-up. For Cox regression analysis, MNA, BMI, serum albumin, C-reactive protein (CRP), and serum creatinine were entered as continuous variables. The association between nutritional status and postoperative complications was assessed using cross-tabulation and Fisher’s exact test. Postoperative complications were classified according to the standardized Clavien–Dindo classification system (7). Statistical significance was defined as *p* < 0.05.

## 3 Results

In this study 98 patients were included for analysis, 61 of which were male. Median age was 67 years, ranging from 41 to 84 years. Sixty individuals were 65 years or older. Weight classification was conducted according to the WHO criteria, finding that 2% had a BMI < 18.5 (underweight), while 98% had a BMI ≥ 18.5 (normal weight, overweight, or obesity). The baseline characteristics of the study population are summarized in Table 1.

**TABLE 1:** Patient characteristics.

Characteristics	*n* = 98
Male gender, *n* (%)	61 (62.2)
Age at surgery in years, median (range)	67 (41–84)
Patients ≥ 65 years, *n* (%)	60 (61.2)
BMI < 18.5, *n* (%)	2 (2)
BMI ≥ 18.5, *n* (%)	96 (98)
Histological subtypes of NSCLC, n (%)	
Adenocarcinoma	58 (59.2)
Squamous cell carcinoma	36 (36.7)
Adenosquamous carcinoma	2 (2)
Large cell carcinoma	2 (2)
TNM/UICC-Stage, *n* (%)	
Stage I	44 (44.9)
Stage II	27 (27.6)
Stage III	23 (23.5)
Stage IV	4 (4.1)
Nutritional status (MNA), n (%)	
Well-nourished (MNA ≥ 24)	65 (66.3)
At-risk of malnutrition (MNA 17–23.5)	31 (31.6)
Malnourished (MNA < 17)	2 (2.0)

TNM, Tumor-Node-Metastasis classification; UICC, Union for International Cancer Control.

The majority of NSCLC cases were adenocarcinoma (*n* = 58, 59.2%), followed by squamous cell carcinoma (*n* = 36, 36.7%). Additionally, adenosquamous carcinoma (*n* = 2, 2%) and large cell carcinoma (*n* = 2, 2%) were also observed. Of the patients who took the MNA, 66.3% were classified as well-nourished (MNA score ≥ 24), 31.6% were at risk of malnutrition (MNA score 17–23.5), and 2% were malnourished (MNA score < 17).

As shown in Table 2, Albumin levels in the well-nourished group are fairly comparable in both male and females, reaching desirable values. For patients at risk of malnutrition, levels seem to diverge; however, they may still be considered acceptable. Regarding CRP, values for females tend toward medically unremarkable levels, while for males, CRP is elevated. This observed gender difference vanishes in the group at risk of malnutrition, where both levels are undesirable. Interestingly, maximum recorded CRP levels are much higher in both male groups compared to female patients (130.0 vs. 49.8, 170.0 vs. 56.1). Serum creatinine levels can be considered normal.

**TABLE 2:** Preoperative biomarkers segmented by MNA category and sex.

	MNA category					
	Well-nourished (MNA ≥ 24)		At risk of malnutrition (MNA 17–23.5)		Malnourished (MNA < 17)	
	Male	Female	Male	Female	Male	Female
**Biomarker**	**(*n* = 42)**	**(*n* = 23)**	**(*n* = 18)**	**(*n* = 13)**	**(*n* = 1)**	**(*n* = 1)**
Albumin [g/l]	41.31	42.12	39.79	42.28	43.40	n.a.
Min–Max	34.6–46.9	28.5– 46.3	22.9–45.1	36.2–46.9		
CRP [mg/l]	18.80	6.15	25.15	19.83	4.90	n.a.
Min–Max	0.3–130.0	0.7–49.8	0.8–170.0	0.5–56.1		
Serum creatinine [mg/dl]	1.14	0.77	0.92	0.66	0.92	n.a.
Min–Max	0.59–8.03	0.5–1.14	0.56–1.70	0.47–0.87	–	–

n.a., not available.

**FIGURE 1:**
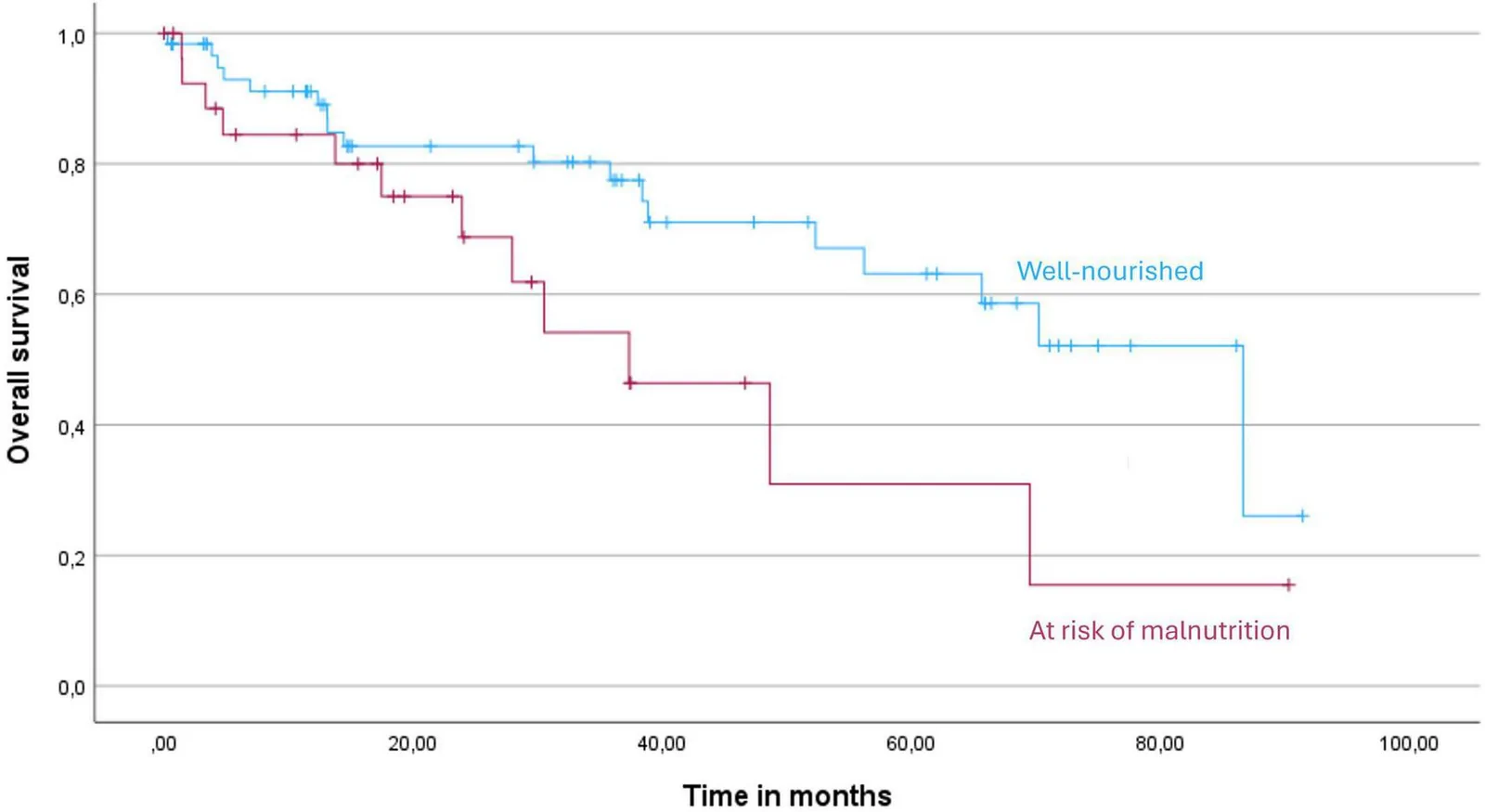
Kaplan–Meier curves showing overall survival (OS) stratified by nutritional status. Differences between groups were assessed using the log-rank test. Tick marks indicate censored observations.

Four patients exhibiting UICC Stage IV were excluded from analysis to prevent bias. The median follow-up period of the study cohort was 53 months (IQR: 29.3–74.0 months). During follow-up, 32 death events occurred in the survival cohort (*n* = 94). Overall survival for the groups of well-nourished patients and those at risk of malnutrition is presented in Figure 1. Median survival was 86.557 ( ± 12.275) months for the well-nourished group and 37.311 ( ± 8.464) months for the patients at risk of malnutrition. Log Rank test reveals a statistically significant difference in survival (χ^2^ = 4.340, df = 1, *p* = 0.037). In patients at risk of malnutrition, 1-year OS was 67.86%, and 5-year OS was 7.14%. In contrast, well-nourished patients showed a 1-year OS of 73.77%, and a 5-year OS of 26.22%.

**FIGURE 2:**
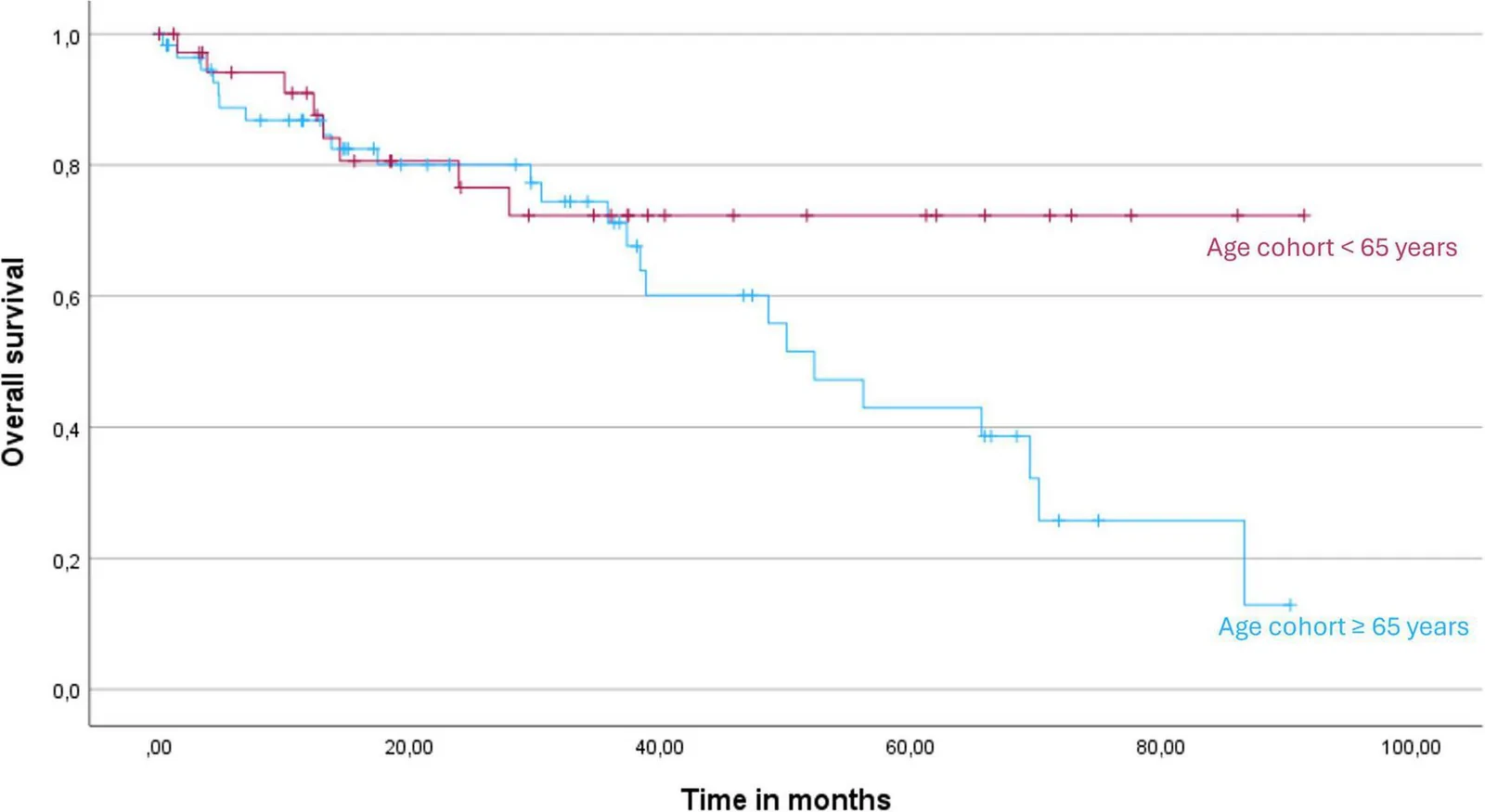
Kaplan–Meier curves showing overall survival (OS) stratified by age group. Differences between groups were assessed using the log-rank test. Tick marks indicate censored observations.

Figure 2 shows overall survival comparing patients at or above the age of 65 to younger individuals. Median survival was 52.051 ± 4.874 months in the age group 65 and older. For the younger group more than 70% survived until the time of the last follow-up, and mean survival is 70.053 ± 6.478 months. Log Rank assessment did not yield a statistically significant result (χ^2^ = 2.857, df = 1, *p* = 0.091); however, the *p* < 0.1 may warrant further investigation in the future. In patients 65 or older, 1-year OS was 69.49%, and 5-year OS was 16.95%. For the younger patients, 1-year OS was 72.90%, and 5-year OS was 21.60%.

For Cox regression analysis, both the Mini-Nutritional Assessment (MNA) and Body Mass Index (BMI) were evaluated as continuous variables to assess their impact on survival outcomes. The remaining independent variables were albumin, CRP, and serum creatinine levels. All predictors were added in a single block. Omnibus test for coefficients shows adequate model fit (χ^2^ = 29.599, df = 5, *p* < 0.001). In the first step, the total sample was assessed. MNA score and serum creatinine level showed the strongest associations with overall survival. Patients with higher MNA scores showed a lower mortality risk (HR = 0.214, 95% CI 0.060–0.765, *p* = 0.018). On the other hand, serum creatinine levels, on average, doubled patients’ risk, with a HR of 2.200 (95% CI 1.146–4.224, *p* = 0.018). CRP showed a modest, although not statistically significant, association with overall survival (HR = 1.019, 95% CI 0.999–1.040, *p* = 0.059). Both BMI and albumin did not show a significant impact in the total sample, as shown in Table 3. Similar results were observed in patients aged ≥ 65 years (model fit: χ^2^ = 46.470, df = 5, *p* < 0.001); however, evidence for an influence of BMI and CRP reaches statistically significant levels in this subgroup.

**TABLE 3:** Cox regression analysis for survival.

Variable	Exp (β) (95% CI)	*p*-value
MNA		
Total sample	0.214 (0.060–0.765)	0.018
Age group ≥ 65	0.068 (0.015–0.319)	< 0.001
BMI		
Total sample	1.817 (0.891–3.706)	0.100
Age group ≥ 65	4.908 (1.977–12.183)	< 0.001
CRP		
Total sample	1.019 (0.999–1.040)	0.059
Age group ≥ 65	1.063 (1.023–1.104)	0.002
Albumin		
Total sample	0.970 (0.878–1.073)	0.559
Age group ≥ 65	0.985 (0.893–1.087)	0.771
Serum creatinine		
Total sample	2.200 (1.146–4.224)	0.018
Age group ≥ 65	2.590 (1.181–5.680)	0.018

In total, 33 patients experienced postoperative complications following surgical treatment for non-small cell lung cancer (NSCLC). These complications were classified according to the Clavien-Dindo classification system. Among them, 9 patients (27.3%) developed major complications (Grades III/IV), all of which required surgical or invasive intervention. In addition, one patient (3.0%) succumbed to multi-organ failure (Grade V).

Table 4 shows the distribution of post-surgery complications stratified by the patients’ MNA status. Cross-tabulation analysis did not reveal a significant association between nutritional status and postoperative complications (Fisher’s exact test, *p* = 0.770; Cramer’s V = 0.073).

**TABLE 4:** Post-surgery complications segmented by MNA status.

Nutritional status (MNA)	Post-surgery complications (n)	No complications (n)
Well-nourished (MNA ≥ 24)	20	45
At risk of malnutrition (MNA 17–23.5)	11	20
Malnourished (MNA < 17)	1	1

## 4 Discussion

### 4.1 Theoretical implications

As our results indicate, well-nourished NSCLC patients exhibit improved survival outcomes, and vice versa. This finding is consistent with extant literature reporting worse OS in patients with poorer nutritional status (8, 9). In this regard, baseline nutritional parameters including weight loss and inflammation markers have been found to correlate adversely with prognosis in advanced NSCLC cases (10).

We contribute to the growing body of evidence by corroborating that nutritional status plays a vital role in the prognosis of elderly NSCLC patients. More specifically, the broader range of information captured by MNA led to a superior prognostic model, indicating that BMI alone may be insufficient to describe the dietary state. Higher MNA scores, i.e., indication for being well-nourished, were associated with a lower mortality risk (HR = 0.214, *p* = 0.018). This effect was even more pronounced in elderly patients (HR = 0.068, *p* < 0.001), underscoring the relevance of good nutrition in this group.

In patients aged 65 years and older, some patient-reported and behavioral characteristics were found to be linked to overall survival. Particularly, inadequate fluid intake and poor self-perceived general health were associated with a marked increase in mortality risk. These findings emphasize that beyond anthropometric measures (weight or BMI), non-anthropometric characteristics may provide additional prognostic information in elderly NSCLC patients. Chronic insufficient fluid intake is common in elderly patients due to age-related reduction in thirst and other factors (11). Inadequate hydration can lead to dehydration, which impairs cardiovascular and renal function, contributes to electrolyte imbalances, and diminishes clearance of bodily waste. In older cancer patients, even mild dehydration is associated with adverse outcomes including longer hospital stays, higher readmission rates, and increased mortality. As poor hydration may also signal frailty or cognitive decline, MNA’s inclusion of fluid intake flags patients who are at risk of dehydration-related complications and overall frailty (12, 13).

Similarly, poor self-rated health integrates physical, functional, and psychological aspects and has repeatedly been shown to be an independent predictor of mortality in both community-dwelling elderly and older cancer patients. Negative health perception often reflects underlying comorbidities or functional decline and may influence treatment adherence and resilience. Conversely, a better self-perceived health status is associated with higher functional reserve and improved outcomes (14, 15).

In line with recent research, we assessed the role of elevated CRP levels for patient survival. Indeed, we find the inflammatory marker to be associated with overall survival. This finding illustrates previous reports documenting substantially increased hazard of death in individuals with very high CRP levels 2.6-fold increase, (16), suggesting that systemic inflammation may impact cancer prognosis and potentially interact with nutritional status. While our results showed stronger evidence for an association with increased mortality in elderly patients (6.3% increase in mortality risk, *p* = 0.002), CRP also showed a modest trend toward increased mortality in the overall sample (1.9%, *p* = 0.059), warranting further investigation. Lastly, serum creatinine should be considered for an estimation of patient survival in all age groups, with a rather substantial risk increase of 120% (*p* = 0.018) for the overall sample and an even more pronounced 159% in the age group above 65 (*p* = 0.018).

We did not find an effect of serum albumin levels on patient survival; however, this null result likely stems from the fact that our sample was located within the reference range of 35–55 g/L, provided by the site’s laboratory. Earlier publications show that patients with normal albumin levels have significantly better survival rates, potentially even halving the risk (HR = 0.50) (16). Further, we checked for potential differences in survival due to patients’ age although Kaplan-Meier curves did not differ significantly. We observe a slight trend for increased OS in younger patients (*p* = 0.091). Hence, age alone may not be considered a sufficient predictor for survival.

Overall, our findings highlight the importance of employing comprehensive screening tools such as the MNA to amend the rather narrow information provided by single measures such as BMI.

### 4.2 Clinical implications and future directions

Nutritional status was strongly associated with survival in NSCLC patients, particularly in elderly patients. Additionally, poor nutritional status has been consistently linked to higher rates of postoperative complications in NSCLC patients undergoing surgery. Although our cohort could not replicate these findings, multiple studies report increased postoperative complications, prolonged hospital stays, and higher in-hospital mortality (8, 17). Mechanistically, malnutrition impairs immune function, wound healing, and physiological reserve, thereby increasing susceptibility to infections and delayed recovery. In thoracic surgery, these effects may translate into respiratory complications, prolonged air leaks, or septic events, particularly in patients with concurrent sarcopenia (18). Evidence from other surgical fields further supports malnutrition as a universal risk factor for adverse postoperative outcomes. Consequently, current clinical guidelines recommend routine nutritional screening and targeted preoperative nutritional interventions (19).

Clinicians benefit from employing MNA as a concise tool to assess nutritional status. This index may be complemented with CRP and serum albumin evaluation to provide a comprehensive prognostic value. While elevated CRP levels diminish chances of survival, albumin levels within the reference range appeared to be associated with a protective effect.

### 4.3 Limitations

As with all scientific studies, a number of limitations need to be addressed. First, our sample did not include enough observations for the malnourished category to include them in formal analysis. In addition, the multivariable model did not include several potentially relevant clinical factors, such as comorbidities, smoking history, tumor stage, performance status, surgical procedure, or adjuvant therapy, which may have influenced survival outcomes. Second, the data were collected in Germany, and potential unobserved confounders warrant caution when applying our findings to different settings. Third, the study was designed as a single-center approach, which may have introduced observational bias. Prospective multicenter studies are required to further corroborate our early insights.

## 5 Conclusion

The work at hand offers exploratory insights into the impact of preoperative nutritional status on NSCLC patients’ survival outcomes after undergoing lung resection surgery. Nearly one-third of patients were identified as being at risk of malnutrition and exhibited poorer long-term survival. The Mini Nutritional Assessment (MNA) was independently associated with overall survival, suggesting its clinical value beyond conventional measures such as BMI. Particularly in elderly NSCLC patients, nutritional screening may represent a valuable addition to standard perioperative care, allowing early identification of high-risk patients and supporting improved clinical management.

## Data Availability

The original contributions presented in this study are included in the article/supplementary material, further inquiries can be directed to the corresponding author.
