# Correction: Malnutrition assessed by the mini nutritional assessment is associated with overall survival in patients with resectable non–small cell lung cancer

**DOI:** 10.3389/fnut.2026.1960385

**Published:** 2026-08-20

**Authors:** Denis I. Trufa, Koblandy Khamitov, Lena Kopplin, Horia Sirbu

**Affiliations:** 1 Department of Thoracic Surgery, Universitätsklinikum Erlangen, Erlangen, Germany; 2 Faculty of Medicine, Friedrich-Alexander-Universität Erlangen-Nürnberg, Erlangen, Germany

**Keywords:** malnutrition, mini nutritional assessment, non–small cell lung cancer, overall survival, prognosis, pulmonary resection

In the original published article, the Acknowledgements section was inadvertently omitted.

The correct **Acknowledgements** statement should read as

The authors would like to thank Tom Börstler for his critical review of the manuscript and his valuable support.

The original version of this article has been updated.

